# Antioxidant Activity, Total Polyphenol Content, and Mineral Composition of Milk Beverages Fortified with Spice Mixtures (Clove, Cinnamon, and Turmeric) and Natural Sweeteners (Erythritol and Stevia): Evidence of Synergistic or Antagonistic Effects of Compounds

**DOI:** 10.3390/ijms26188813

**Published:** 2025-09-10

**Authors:** Karolina Rak, Joanna Kolniak-Ostek, Robert Gajda, Katarzyna Marcinkiewicz, Agnieszka Nemś, Ewa Raczkowska

**Affiliations:** 1 Department of Human Nutrition, Faculty of Biotechnology and Food Science, Wrocław University of Environmental and Life Sciences, 51-630 Wrocław, Poland; robert.gajda@upwr.edu.pl (R.G.); katarzyna.marcinkiewicz@upwr.edu.pl (K.M.); ewa.raczkowska@upwr.edu.pl (E.R.); 2 Department of Fruit, Vegetable and Plant Nutraceutical Technology, Faculty of Biotechnology and Food Sciences, Wrocław University of Environmental and Life Sciences, 51-630 Wrocław, Poland; joanna.kolniak-ostek@upwr.edu.pl; 3 Department of Food Storage and Technology, Faculty of Biotechnology and Food Science, Wrocław University of Environmental and Life Sciences, 51-630 Wrocław, Poland; agnieszka.nems@upwr.edu.pl

**Keywords:** functional food, polyphenols, mineral elements, antioxidant activity, spices, sugar substitutes

## Abstract

Extensive research is underway on a variety of functional foods that support consumer health. A promising combination is milk (a drink with high nutritional value), fortified with spices (naturally rich in antioxidant compounds) and supplemented with low-calorie, health-promoting sweeteners. The aim was to analyze the antioxidant activity (AA), total polyphenol content (TPC), and mineral composition of milk beverages fortified with a mixture of spices and sweeteners and to verify the interactions between these ingredients. Twenty-four variants of milk drinks were prepared with the addition of three types of spice mixtures (1:1) of clove (Cl), cinnamon (Ci), and turmeric (Tu) with the shares of 2.5%, 5%, 7.5%, and 10%, and two types of sweeteners—erythritol (E) and stevia (S)—as well as six control samples, including three 10% aqueous solutions of spice mixtures. AA was measured using the ABTS, FRAP, and DPPH methods. TPC was determined using the Folin–Ciocalteu method. Mineral content (Ca, Mg, Fe, Cu, and Zn) was assessed using the FAAS/FAES method. The highest AA was demonstrated for beverages with Cl-Ci and Cl-Tu, whereas the highest TPC was found in those with Cl-Tu. AA and TPC values increased with the increase in the share of spices in the beverages, and both measurements were significantly higher in beverages with erythritol compared to those with stevia. Despite the 4–5 times lower TPC, 3–8 times higher AA was demonstrated in beverages with the addition of 10% spice mixtures than their corresponding control samples (aqueous solutions with the addition of 10% spice mixtures), which suggests the great role of interactions between nutrients in food. For beverages with 10% Cl-Ci/Cl-Tu mixtures, significantly higher ABTS, FRAP, and DPPH were observed than would result from the simple sum of AA of the components (synergistic effect). The opposite (antagonistic) effect was observed for beverages with Ci-Tu. AA was positively correlated with the content of Fe, Mg, and Cu; negatively with Ca; and not correlated with Zn. The results suggest that the most health-promoting properties are exhibited by the milk drink with the addition of 10% Cl-Tu and erythritol, demonstrating the highest AA and TPC, the strongest synergistic effect of the components, and the highest content of Mg and Zn. This study highlights the importance of carefully selecting and combining ingredients to maximize the antioxidant properties of functional beverages. However, further research is needed to expand knowledge on this issue.

## 1. Introduction

Functional foods are generally considered as foodstuffs that provide claimed beneficial health effects on the human body beyond those resulting from the basic nutritional value [1]. In recent years, due to rising nutritional and lifestyle awareness, consumer interest in functional food and health-promoting sugar substitutes has been growing steadily, which is reflected in the dynamic development of these branches of the food market [2,3].

Extensive research is underway on a variety of functional foods that support consumer health. Spices can be used as valuable functional food additives. Being a naturally rich source of bioactive compounds such as vitamins, minerals, phytochemicals (i.e. polyphenols, phytosterols, terpenoids, alkaloids, and glycosides), essential oils, and many others [4], they express a number of multidirectional health-promoting properties, including antimicrobial, antioxidant, anti-inflammatory, immunomodulatory, anti-cancer, and anti-hypertensive effects; improvement of glucose and lipid profiles; and anthropometric measurements, as well as positively affecting cognitive abilities and mood [5,6,7]. Their beneficial properties have been used in the prevention and even as supportive elements of therapy in obesity, diabetes, hypertension, polycystic ovary syndrome (PCOS), nonalcoholic fatty liver disease (NAFLD), hyperlipidemia, metabolic syndrome (MetS), and Hashimoto’s thyroiditis, as demonstrated in many studies [4,7,8].

Clove (Cl), cinnamon (Ci), and turmeric (Tu) may have significant potential in the design of functional foods, as they have, according to the ORAC scale, some of the highest antioxidant activity among spices (290.283, 131.420, and 127.068 µM Tx/100 g, respectively) [9]. Their consumption has been shown to be associated with improvements in metabolic, anthropometric, mood-related, and cognitive parameters [10,11,12,13,14,15]. Similarly, erythritol (E) and stevia (S), as safe and well-tolerated natural sugar substitutes [16,17,18], can be successfully used in functional products, especially for people with overweight, obesity, or diabetes, as they provide almost no energy (<0.4 kcal/g of erythritol and 0 kcal/g of stevia) [19,20], can modulate satiety and appetite [21], do not increase postprandial glucose [22], and have a glycemic index equal to 0 [23]. Moreover, they express anti-caries properties [24,25]. Erythritol, a sugar alcohol naturally found in many fruits, is a low-intensity sweetener with a sweetness of 50–70% compared to saccharose [26], and its acceptable daily intake (ADI) is established by the European Food Safety Authority (EFSA) at 500 mg/kg of body weight [17]. Stevia, a high-intensity sweetener produced from *Stevia rebaudiana* leaves, is 250–300 times sweeter compared to saccharose [20], and an ADI of steviol glycosides of 4 mg/kg of body weight is established by EFSA [18].

Milk and other dairy products can be considered a good functional food matrix due to their high nutritional value. They are a rich source of high-quality protein, calcium, phosphorus, potassium, iodine, and vitamins B2 and B12 [27]. They have also been shown to be good carriers of functionally beneficial bioactive plant compounds contained in herbs and spices [28,29]. Milk proteins bind to polyphenols, improving their gastrointestinal stability, protecting them from oxidative degradation during the digestive process and resulting in their higher bioaccessibility and bioavailability [30,31,32,33,34]. Milk fat combined with milk proteins has also been shown to increase the bioavailability of polyphenols after digestion and plasma antioxidant activity [35]. Although milk fat alone has the opposite effect on plasma antioxidant activity [36], it plays a beneficial functional role related to the ability to “capture” polyphenols and prolong their transport through the gastrointestinal tract, increasing their transport to the large intestine, where they support the development of beneficial gut microflora [37]. Interestingly, polyphenols can bind to digestive proteins (enzymes), leading to limited digestion of macronutrients (especially lipids and digestible carbohydrates) and reduced nutritional value of food, while at the same time leading to an increased functional effect in the control of obesity, diabetes, and metabolic syndrome [33,37,38].

It has been demonstrated that not only macronutrients but also minerals interact with polyphenols. However, knowledge on this topic is limited, and the results presented in the literature are often inconsistent. The antioxidant activity of polyphenol–mineral complexes depends on the molecular structure and electrical charge distribution of phenolic compounds (e.g., the presence and number of hydroxyl and carboxyl groups, double bond conjugation, and stabilization of the electronic charge distribution), metal properties (e.g., oxidation state and redox potential), as well as the interdependence of the above features. Therefore, the final antioxidant effect (enhancement or reduction) is difficult to predict, which is confirmed by many documented cases in which the same polyphenol–metal complexes showed different effects [39]. While chelation of toxic elements such as lead, cadmium, and mercury by polyphenols increases the functional properties of foods by reducing their absorption, concentration in the blood, and accumulation in internal organs [40], binding polyphenols with other metal ions, such as iron and zinc, may limit their absorption [41,42], reducing the nutritional value of food. It has been reported that metal ions (e.g., silver, copper, and zinc), when bound to a phenolic compound, enhance its natural activity, including antimicrobial, antioxidant, and anti-inflammatory effects, and also trigger additional activities such as collagen synthesis and fibroblast activity, demonstrating their synergistic interaction with functional potential [43].

The literature also provides a wealth of descriptions of possible interactions between polyphenols, both synergistic and antagonistic, using combinations of various phenolic compounds in various proportions [44,45,46]. Researchers emphasize, however, that there is still a lack of information on this topic, and the multitude of factors modifying interactions, such as the combined ratio or concentration of antioxidants, food matrix, temperature, pH, storage conditions, light and oxygen availability, and processing and cooking method [47,48,49,50,51], makes it difficult to predict the final effect. Despite this, most studies indicate that polyphenols may be more bioavailable when consumed together with other polyphenols than when taken separately in supplement form [38]. Hence, combining polyphenols from different spices seems to be the right direction of research on functional foods. The fortification of the milk with spices can improve not only its health-promoting value but also the sensory and physicochemical properties of the final product, including its color, smell, taste, moisture, texture, and appearance [52,53,54,55]. Moreover, due to their antimicrobial and antioxidant activity, polyphenols act as natural preservatives in these products [56]. The successful application of various herbs and spices in multiple forms (e.g., fresh, dried and ground, powder, extracts, essential oils, etc.) into dairy products such as milk, yoghurts, ice cream, and various types of cheeses has been well documented [28,29,57].

The aim of this study was to assess the health-promoting potential of milk beverages fortified with various spice mixtures (ground clove, cinnamon, and turmeric) with different proportion in the product (2.5%, 5%, 7.5%, and 10%) and with the addition of natural sweeteners (erythritol and stevia). The antioxidant activity (AA), total polyphenol content (TPC), and mineral composition were investigated, and the interactions between compounds in the beverages were also explored.

## 2. Results

### 2.1. Antioxidant Capacity

**Figure 1:**
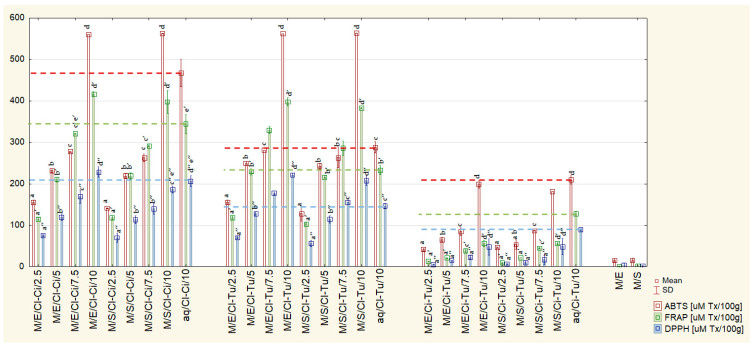
Antioxidant capacity (ABTS, FRAP, and DPPH) of the tested milk beverages. M—milk; E—erythritol; S—stevia; Cl-Ci—clove–cinnamon; Cl-Tu—clove–turmeric; Ci-Tu—cinnamon–turmeric; 2.5, 5, 7.5, 10—share of spice mixture [%]; aq—aqueous solutions of spice mixtures; control samples: aq/Cl-Ci/10, aq/Cl-Tu/10, aq/Ci-Tu/10, M/E, M/S. - - - Marker of ABTS, FRAP, and DPPH values of control samples; small letters indicate statistically not significant differences between samples: a–d for ABTS values, a’–e’ for FRAP values, and a″–e″ for DPPH values; *p* > 0.05. In the Supplementary Materials, all significant differences between pairs of tested beverages and control samples (Supplementary Table S1A–I) are presented.

Table 1 presents the results of the three-way ANOVA demonstrating the effect of the type of spice mixture, its share [%], and the type of sweetener, as well as their interactions, on the antioxidant capacity (ABTS, FRAP, and DPPH) of the tested beverages. All main effects and their interactions were statistically significant for all three parameters corresponding to antioxidant capacity, except for two interactions—spice mixture * sweetener and share of spice mixture [%] * sweetener—which were significant only for FRAP and DPPH, but not for ABTS. Table 2 presents the results of Tukey’s post hoc test for main effects. The lowest antioxidant capacity was shown for the Ci-Tu mixture, while the Cl-Ci and Cl-Tu mixtures exhibited 3–8 times significantly higher activity, without any significant differences between them. With the increase in the share of spices, the antioxidant capacity increased significantly for all measurements—ABTS, FRAP, and DPPH. Beverages with the addition of erythritol had significantly higher antioxidant capacity than those with stevia. Figure 1 presents the means and standard deviations of ABTS, FRAP, and DPPH for all tested beverages and control samples. Due to numerous statistically significant differences, non-significant ones are marked in the figure. In the Supplementary Materials, all significant differences between pairs of tested beverages and control samples (Supplementary Table S1A–I) are presented. Compared to the corresponding control sample aq/Cl-Ci/10, the beverages with the addition of 10% Cl-Ci exhibited significantly higher antioxidant capacity (by 15.5–20.8%, depending on the method). Similarly, compared to the corresponding control sample aq/Cl-Tu/10, the beverages with the addition of 10% Cl-Tu and 7.5% Cl-Tu exhibited significantly higher antioxidant capacity (by 42.1–92.5% and 5.8–41.6%, respectively). Conversely, those with the addition of 10% Ci-Tu had significantly lower activity than their corresponding control sample aq/Ci-Tu/10. The control samples of sweetened milk M/E and M/S exhibited very low antioxidant capacity, ranging from 0.73 ± 1.13 µM Tx/100 g (FRAP) to 14.43 ± 3.46 µM Tx/100 g (ABTS) and from 1.36 ± 1.61 µM Tx/100 g (DPPH) to 15.45 ± 5.08 (ABTS), respectively.

**Figure 2:**
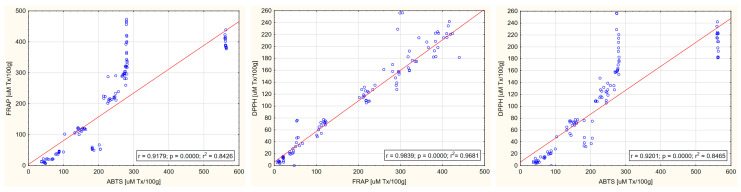
Correlation between FRAP, ABTS, and DPPH of the tested milk beverages.

Figure 2 presents correlations between FRAP, ABTS, and DPPH of the tested beverages. The correlation of all three parameters of antioxidant activity was strongly positive (0.92 < r < 0.98) and statistically significant (*p* < 0.000). The highest coefficient of determination r^2^ was demonstrated for the DPPH:FRAP correlation (r^2^ = 0.968).

### 2.2. Total Polyphenol Content (TPC)

**Figure 3:**
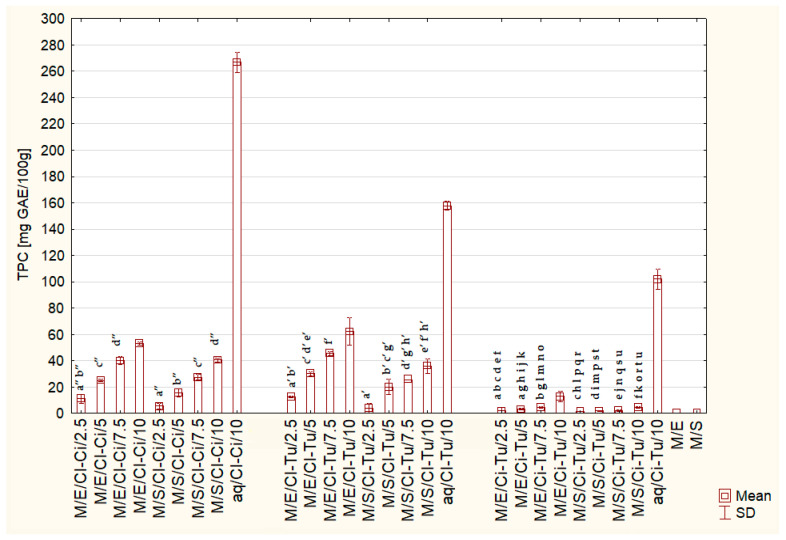
Total polyphenol content (TPC) of the tested milk beverages. M—milk; E—erythritol; S—stevia; Cl-Ci—clove–cinnamon; Cl-Tu—clove–turmeric; Ci-Tu—cinnamon–turmeric; 2.5, 5, 7.5, 10—share of spice mixture [%]; aq—aqueous solutions of spice mixtures; control samples: M/E, M/S, aq/Cl-Ci/10, aq/Cl-Tu/10, aq/Ci-Tu/10; small letters indicate statistically not significant differences between samples: a″–d″ for Cl-Ci mixture, a′–h′ for Cl-Tu mixture, a–u for Ci-Tu mixture; *p* > 0.05. In the Supplementary Materials, all significant differences between pairs of the tested beverages and control samples (Supplementary Table S2A–C) are presented.

Table 3 presents the results of the three-way ANOVA demonstrating the effect of the type of spice mixture, its share [%], and the type of sweetener, as well as their interactions on the total polyphenol content of the tested beverages. All main effects and their interactions were statistically significant (*p* < 0.000), except for the interaction spice mixture * share of spice mixture [%] * sweetener, the significance of which was shown to be borderline (*p* = 0.058). Table 4 presents the results of Tukey’s post hoc test for main effects. TPC in beverages with the addition of Cl-Tu was the highest, while in those with the addition of Ci-Tu, it was the lowest (29.49 mg vs. 3.94 GAE/100 g, *p* < 0.001). With the increase in the share of spices, TPC increased significantly. Beverages sweetened with erythritol were characterized by significantly higher TPC than those sweetened with stevia. Figure 3 presents the means and standard deviations of TPC for all tested beverages and control samples. Only the most important statistically significant differences are marked. In the Supplementary Materials, all significant differences between pairs of tested beverages and control samples (Supplementary Table S2A–C) are presented. The control samples aq/Cl-Ci/10, aq/Cl-Tu/10, and aq/Ci-Tu/10 exhibited significantly higher TPC than the corresponding tested beverages M/Cl-Ci/10, M/Cl-Tu/10, and M/Ci-Tu/10 (266.97 vs. 46.76, 157.88 vs. 49.11, 102.01 vs. 8.52 mg GEA/100 g, *p* < 0.001, respectively). The control samples of sweetened milk M/E and M/S exhibited TPC close to 0 (0.11 ± 0.22 mg GEA /100 g) or 0.00 ± 0.00, respectively.

**Figure 4:**
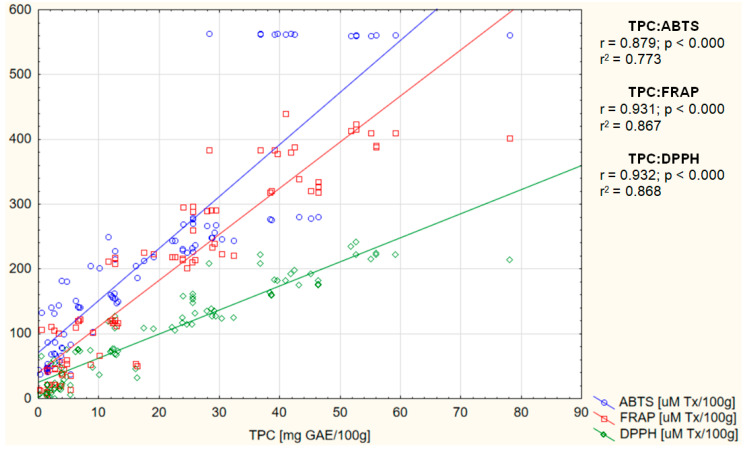
Correlation between total polyphenol content (TPC) and antioxidant activity (FRAP, ABTS, and DPPH) of the tested milk beverages.

Figure 4 presents correlations between TPC and FRAP, ABTS, and DPPH of the tested beverages. The correlation of TPC with all three parameters of antioxidant activity was strongly positive (0.0.88 < r < 0.93) and statistically significant (*p* < 0.000). The highest coefficient of determination r^2^ was demonstrated for TPC:DPPH (r^2^ = 0.868).

### 2.3. Mineral Element Content

Table 5 presents the results of the three-way ANOVA demonstrating the effect of the type of spice mixture, its share [%], and the type of sweetener, as well as their interactions, on the content of mineral elements (Ca, Mg, Fe, Zn, and Cu) of the tested beverages. All main effects and their interactions were statistically significant (*p* < 0.000). Table 6 presents the results of Tukey’s post hoc test for the main effects. The highest content of Ca was found in beverages with the addition of Ci-Tu (111.61 ± 5.30 mg/100 g, *p* = 0.000), higher in those with stevia than erythritol (110.73 vs. 102.76 mg/100 g, *p* = 0.000). With the increase in the share of spices, the content of Ca decreased significantly. The highest content of Mg and Zn was found in beverages with the addition of Cl-Tu (22.73 ± 5.46 mg/100 g and 285.54 ± 15.73 µg/100 g, *p* = 0.000, respectively), higher in those with stevia than erythritol (21.70 vs. 19.60 mg/100 g and 276.81 vs. 272.58 µg/100 g, *p* = 0.000, respectively). With the increase in the share of spices, the content of Mg and Zn increased significantly. The highest content of Fe and Cu was found in beverages with the addition of Cl-Ci (0.785 ± 0.085 mg/100 g and 79.87 ± 30.05 µg/100 g, *p* = 0.000, respectively), higher in those with erythritol than stevia (0.736 vs. 0.715 mg/100 g and 79.07 vs. 38.87 µg/100 g, *p* = 0.000, respectively). With the increase in the share of spices, the content of Fe and Cu increased significantly.

**Figure 5:**
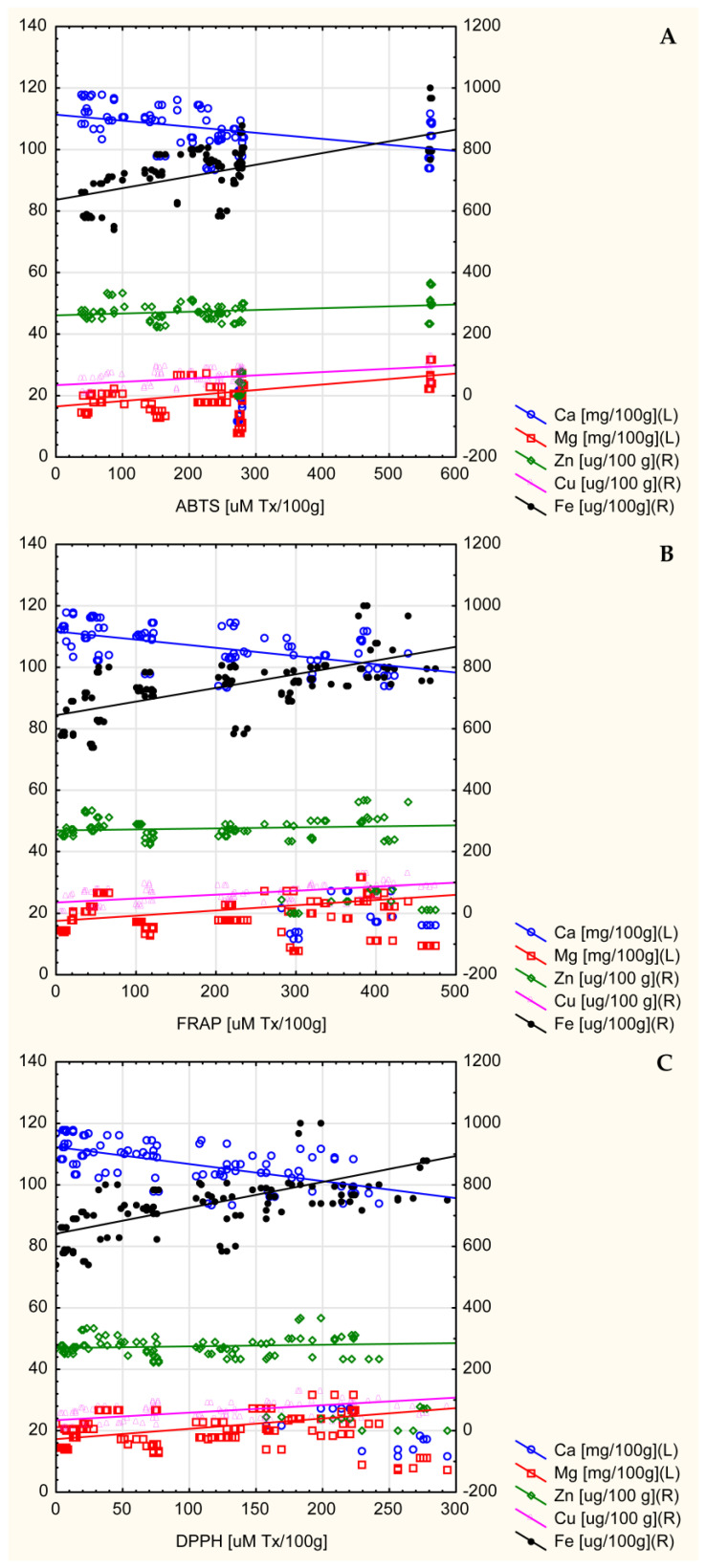
Correlation between antioxidant activity—ABTS (**A**), FRAP (**B**), DPPH (**C**), and mineral elements (Ca, Mg, Fe, Zn, and Cu) in the tested beverages.

Figure 5A–C and Table 7 present correlations between FRAP, ABTS, and DPPH and the content of mineral elements (Ca, Mg, Fe, Zn, and Cu) of the tested beverages. All three parameters of antioxidant activity were moderately positively correlated with the content of Mg and Cu and strongly positively correlated with Fe (*p* < 0.000). A moderate negative correlation was found for Ca, but no statistically significant correlation for Zn.

### 2.4. Additive, Synergistic, and Antagonistic Effect of Compounds

**Figure 6:**
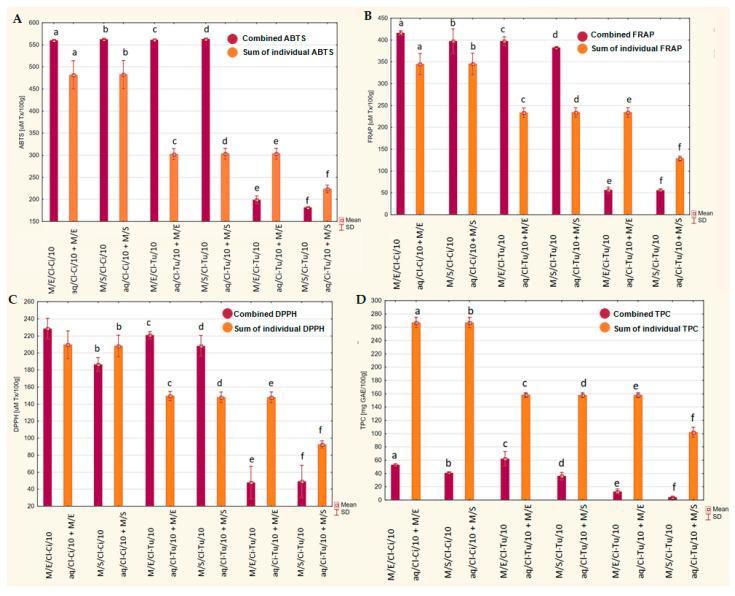
Additive, synergistic, and antagonistic effect of compounds of the tested beverages on ABTS (**A**), FRAP (**B**), DPPH (**C**), and TPC (**D**). Combined and summed mean value ± SD are presented. a–f—statistically significant differences in pairs, *p* < 0.05. Combined values are mean values of ABTS, FRAP, DPPH, and TPC of the tested milk beverages with the addition of sweetener and the 10% spice mixture. Summed values are the simple sum of mean values of ABTS, FRAP, DPPH, and TPC of aqueous control solutions of 10% spice mixtures and mean values of ABTS, FRAP, DPPH, and TPC of milk matrix sweetened with erythritol or stevia.

Figure 6 presents the additive, synergistic, and antagonistic effects of compounds of the tested beverages on ABTS, FRAP, DPPH, and TPC. Combined and summed mean values ± SD are presented. The combined values are mean values of ABTS, FRAP, DPPH, and TPC of tested milk beverages with the addition of sweetener and the 10% spice mixture and should be considered as a result of synergism or antagonism of compounds contained in the tested beverages. The summed values are the simple sum of mean values of ABTS, FRAP, DPPH, and TPC of aqueous control solutions of the 10% spice mixture and mean values of ABTS, FRAP, DPPH, and TPC of the milk matrix sweetened with erythritol or stevia and should be considered the simple additive effect of compounds contained in these substrates. For Cl-Ci (a-b) and Cl-Tu (c-d) mixtures, irrespective of the type of sweetener, a statistically significant synergistic effect for ABTS, FRAP, and DPPH (except for Cl-Ci in the case of DPPH) was observed (Figure 6A–C). Moreover, for the Cl-Tu mixture, compared to Cl-Ci, greater synergistic effects were demonstrated, with increases of 85.5–85.6% vs. 16.3–16.5% (ABTS), 63.3–70.2% vs. 15.0–20.6% (FRAP), and 40.8–47.8% (DPPH), respectively. The positive effect was slightly higher for beverages with the addition of erythritol compared to stevia. Conversely, for beverages with the addition of the Ci-Tu mixture (e-f), antagonistic effects for all parameters of antioxidant activity were observed—a decrease of 18.9–34.4% (ABTS), 56.5–76.1% (FRAP), and 47.1–67.8% (DPPH). The negative effect was slightly higher for beverages with the addition of erythritol compared to stevia.

Antagonistic effects for TPC for all three types of spice mixtures, irrespective of the type of sweetener, were demonstrated (Figure 6D). The greatest antagonistic effect was shown for Ci-Tu, a decrease of 91.9–95.8%, followed by Cl-Ci, a decrease of 80.2–84.8%, and Cl-Tu, the smallest decrease of 60.6–77.2%.

## 3. Discussion

In this study, beverages with the addition of Cl-Ci and Cl-Tu mixtures exhibited the highest antioxidant capacity and TPC, significantly higher than those with the Ci-Tu mixture (Table 2 and Table 4). This finding is consistent with data presented by the ORAC scale on the antioxidant activity of spices, which demonstrates higher antioxidant activity of clove (290,283 µM Tx/100 g) compared to cinnamon (131,420 µM Tx/100 g) and turmeric (127,068 µM Tx/100 g) [9]. Moreover, both antioxidant activity and TPC increased with the increase in the share of spices in the beverages, which confirms that the dominant source of this activity is spices rich in polyphenols with strong antioxidant properties [58,59]. The tested beverages with the addition of erythritol had significantly higher antioxidant capacity (Table 2) and TPC (Table 4) than those with stevia. This is probably the result of a much lower share of stevia than erythritol in the final product weight (0.0083% vs. 0.05%) due to its very high sweetness (300 times vs. 0.5 times compared to saccharose) [20,26] and also indicates the antioxidant properties of erythritol, which is consistent with the results of other studies [60,61].

In this study, higher antioxidant activity was observed in the tested beverages with a 10% addition of spice mixtures compared to their corresponding aqueous control samples (Figure 1), even though the control samples had significantly higher TPC (Figure 3). This suggests synergy between the ingredients of the tested beverages—spices, milk, and sweeteners. Moreover, greater differences in antioxidant activity between beverages with the addition of Cl-Ci/Cl-Tu vs. Ci-Tu (3–8 times, Table 2), compared to the differences between pure spices—Cl vs. Ci and Cl vs. Tu (2 times) [9], were observed. This similarly suggests synergistic interactions between compounds in beverages with the addition of clove. These synergistic effects are clearly demonstrated in Figure 6A–C(a–d), when comparing combined and summed values of ABTS, FRAP, and DPPH. The combination of spices, sweeteners, and milk in the tested beverages exhibited significantly higher antioxidant activity than spices and sweetened milk separately. This finding supports Wang et al.’s (2011) conclusion that combining foods from different categories is more likely to result in synergistic antioxidant activity than combining foods within the same group [62]. According to Bayram and Decker (2023), synergistic antioxidant interactions can be explained by (1) the regeneration of an oxidized antioxidant by another compound due to redox cycling, (2) the differences in antioxidant partitioning within the food matrix, (3) the combination of free radical scavenging and metal chelating activities, and (4) the formation of additional antioxidant compounds [63]. Other studies have demonstrated that polyphenols can interact with proteins, producing protein–polyphenol complexes, which mostly possess stronger antioxidant potential than the compounds alone [31,32]. Milk proteins have been shown to bind to polyphenols, improving their gastrointestinal stability, protecting them from oxidative degradation during digestion, and resulting in higher bioaccessibility and bioavailability [30]. Similarly, milk fat combined with milk proteins has also been shown to increase the bioavailability of polyphenols after digestion and plasma antioxidant activity [35]. However, it should be emphasized that the interaction effect depends on the type of polyphenol, type of protein, and protein/polyphenol ratio [32,64]. Sun et al. (2022) suggest that ultrasound-assisted alkaline preparation—used in this study—is an effective method of creating such protein–polyphenol conjugates [32], which could explain the above observations.

Interestingly, a greater synergistic effect was observed for beverages with the Cl-Tu mixture than with the Cl-Ci mixture, which suggests a higher potential of turmeric than cinnamon to enhance the antioxidant activity of clove, despite the similar ORAC values of both spices [9]. Similarly, Hossain et al. (2023) demonstrated that the addition of turmeric to a mixture of spices (celery, parsley, thyme, basil, and sage) was responsible for the synergistic antioxidant activity and suggested that this effect may be related to the presence of enolic hydroxyl groups in turmeric [65]. The enolic hydroxyl groups present in curcumin (absent in cinnamaldehyde, the major phenolic compound of cinnamon) can enhance its own antioxidant activity (due to the stabilization of curcumin in the form of an enolic anion through resonance, i.e., the spread of a negative electric charge throughout the structure by a system of conjugated double bonds), and at the same time, can promote the regeneration of phenolic forms of eugenol (the main phenolic compound of clove), resulting in a stronger, synergistic antioxidant effect [66]. Contrary to the synergistic effect in beverages with Cl-Ci and Cl-Tu, beverages with the addition of the Ci-Tu mixture showed the lowest antioxidant activity (Table 2), as well as antagonistic effects and a decrease in all three parameters of antioxidant activity (Figure 6A–C, (e,f)). The negative interactions between cinnamon and turmeric could be explained by competition of the antioxidant compounds in these spices for participation in the reaction with the radical, which causes the faster compound (curcumin from turmeric) to react with the radical first, preventing the slower compound (cinnamaldehyde and catechins from cinnamon) from reacting [67]. On the other hand, cinnamaldehyde is a particularly reactive carbonyl compound that can react with the enolic hydroxyl groups of curcumin via condensation mechanisms (e.g., aldol-type) or form adducts via nucleophilic reactions, which may lead to the degradation of curcumin or its transformation into forms with weaker antioxidant activity [68]. Moreover, in the presence of cinnamaldehyde, the mechanism of mutual regeneration of polyphenols is disturbed, and the enolic hydroxyl groups of curcumin lose the ability to restore the activity of phenols because the aldehyde “captures” curcumin molecules instead of allowing them to regenerate other antioxidants [69]. Finally, instead of an additive or synergistic effect, a mutual weakening of antioxidant activity is observed, which seems consistent with the results of this study—antagonistic interactions in beverages with the addition of Ci-Tu, as well as weaker synergistic interactions in beverages with the addition of Cl-Ci compared to Cl-Tu.

It should, however, be highlighted that many factors can modify antioxidant activity of the product, as well as the synergistic or antagonistic antioxidant effects of compounds, including combined ratio or concentration of antioxidants, food matrix, temperature, pH, storage conditions, light and oxygen availability, and processing and cooking method [47,48,49,50,51]. The same compounds in different conditions can interact in the opposite way; thus, interactive effects are difficult to postulate theoretically [51]. Chen et al. (2021) stated that a larger proportion of hydrophilic components promotes synergistic effects, whereas antagonistic effects generally occur when lipophilic components predominate [51]. This seems to contradict the results of this study, where both synergistic and antagonistic antioxidant effects were observed, even though the main compounds of all three types of spices used in the experiment are lipophilic in nature—eugenol of *Syzygium aromaticum* L. [70], cinnamaldehyde of *Cinnamonum verum* [71], and curcumin of *Curcuma longa* [72].

With the increase in the share of spices, an increase in the content of iron, copper, zinc, and magnesium in the tested beverages was observed, as was a decrease in the content of calcium (Table 6). The results indicate that calcium is of dairy origin (as the share of spices increases, the share of milk, including calcium, decreases), while the main source of the remaining mineral elements is spices, which is consistent with the results of other studies indicating spices as a rich source of not only antioxidant polyphenols but also mineral elements [73,74,75]. Furthermore, a strong positive correlation was demonstrated between the content of iron, copper, and magnesium in the tested beverages and their antioxidant activity, while a negative correlation was shown for calcium and no significant correlation for zinc (Figure 5 and Table 7). The relationships observed in this study are partially consistent with the results presented by other researchers. Ragaee et al. (2006) demonstrated that grains (barley and millet) adapted to growing conditions in the United Arab Emirates, characterized by the highest content of minerals, including calcium, magnesium, zinc, copper, and iron, also showed the highest antioxidant activity compared to those with lower mineral content [76]. Similar results for magnesium were reported by Ciscomani-Larios et al. (2021), who established that magnesium sulfate treatment of snap beans enhanced antioxidant activity, total flavonoids, and total anthocyanins of their edible parts [77]. The literature is, however, inconsistent, as magnesium sulfate was shown by Szentmihályi et al. (2014) to act as a pro-oxidant. The authors also indicated that various magnesium compounds used in the food industry can exhibit different oxidative activities, including antioxidant activity (magnesium citrate and malate), pro-oxidant activity (magnesium gluconate, magnesium polygalacturonate, magnesium oxide, and magnesium sulfate), as well as oxidative neutrality (magnesium chloride) [78]. Inconsistencies in the literature, as well as the dependence of magnesium properties on its chemical form, makes it difficult to draw conclusions about the relationship between magnesium content and antioxidant activity of the final product. There are also ambiguous results regarding the effect of iron and zinc on the antioxidant activity of food. In this study, a strong positive correlation was found between iron content and the antioxidant activity of beverages, while no significant relationship was found for zinc. Conversely, Zago and Oteiza (2001) showed that increased antioxidant activity (in the form of inhibition of lipid oxidation of liposome membranes) was associated with the simultaneous addition of zinc to lipophilic α-tocopherol as well as water-soluble epicatechin [79], suggesting that, regardless of the hydro- or lipophilic properties of polyphenols, their complexes with zinc show a synergistic antioxidant effect. Moreover, in contrast to this study and the study by Zago and Oteiza (2001), Vieira et al. (2013) reported that polyphenol-rich edible *Pleurotus ostreatus* mushrooms enriched with zinc and iron exhibited reduced antioxidant activity compared to those without enrichment [80]. Interestingly, the authors noted that only the values of DPPH radical scavenging activity were reduced, while no differences were observed for antioxidant activity measured using the b-carotene-linoleic acid system, which indicated the way these elements worked. The authors concluded that, due to the complexity of antioxidant mechanisms, it is essential to use multiple methodologies to evaluate their activity, as it is not uncommon to obtain seemingly contradictory findings when assessing antioxidants across various in vitro systems [80]. Thus, in this study, three methods were employed to measure the antioxidant activity of the tested beverages. A strong positive correlation between FRAP, ABTS, and DPPH was shown (Figure 2), as well as between all three measurements of antioxidant activity and TPC (Figure 4). This is consistent with the literature, which also demonstrates strong positive correlation of the examined measurements [51,81,82].

The discrepancies in the literature regarding the antioxidant activity of polyphenol–metal complexes may result not only from differences in antioxidant mechanisms of phenolic compounds and methodological differences but also, or even primarily, from the dual role of metal ions in shaping the antioxidant activity of polyphenols. Chen et al. (2024) explained that metals can either enhance or inhibit antioxidant activity. They may promote activity by redistributing electron density, lowering hydrogen transfer energy, and stabilizing resonance, or reduce it through charge irregularities, covalent bonding, and resonance disruption. The final effect depends on the metal’s properties and its interaction with the ligand’s (polyphenol’s) electronic structure [39].

Due to the multitude of factors determining the antioxidant activity of a food product, further research is necessary to experimentally develop the composition and technological production process of functional beverages in order to achieve the greatest health-promoting effect.

## 4. Materials and Methods

### 4.1. Groceries

The ground spices—clove (*Syzygium aromaticum*—100%; The Union of the Comoros), Ceylon cinnamon (*Cinnamonum verum*—100%; Ceylon), and turmeric (*Curcuma longa*—100%; India) were purchased online from the producer Przyprawomat.pl (Stawiszyn, Poland). The sweeteners—erythritol (erythritol—100%; China) and stevia (steviol glycosides from stevia—97%; Poland)—were also purchased online from Big Nature (Mix Brands, Warszawa, Poland) and Natusweet (Reisenberger Food GmbH, Perchtoldsdorf, Germany), respectively. The matrix for preparing beverages was semi-skimmed (2% fat) UHT cow’s milk (Łaciate, Mlekpol, Łomża, Poland). The characteristics of the nutritional values and polyphenol content of raw materials used in the production of the tested beverages per 100 g are presented in Table 8.

### 4.2. Reagents

Nitric acid, hydrochloric acid, sodium carbonate, ferrous chloride, and Folin–Ciocalteu reagent were purchased from Alchem (Toruń, Poland). Methanol hypergrade for LC-MS, 2,4,6,-tris(2-pirydyl)-s-triazine (TPTZ), 2,2′-azino-bis(3-ethylbenzthiazoline-6-sulfonic acid) (ABTS), potassium peroxodisulfate, 2,2-diphenyl-1-picrylhydrazyl (DPPH), and 6-hydroxy-2,5,7,8-tetramethylchroman-2-carboxylic acid (Trolox) were purchased from Merck (Darmstadt, Germany). All reagents used were of analytical grade.

### 4.3. Preparation of Beverages—Milk Beverages Fortified with Spices and Sweeteners

Twenty-four variants of beverages were prepared, one sample of each variant. Milk was sweetened with either erythritol (E) or stevia (S). The amount of each sweetener used was equivalent to 5 g of saccharose per 200 g of beverage. Taking into account their sweetness compared to saccharose, i.e., 0.5-fold [26] and 300-fold [20], respectively, erythritol constituted 5%, whereas stevia 0.0083% of the final product weight. To achieve such a low share of stevia in the beverage, a stevia solution was first prepared—0.833 g of stevia diluted with milk up to 100 g. Then, 1 g of milk–stevia solution (containing 0.00833 g of stevia) was added to the beverage. Within each sweetener, three variants of two-component spice mixtures—composed of clove (Cl), cinnamon (Ci), and turmeric (Tu) in a 1:1 ratio—were used in different shares (2.5/5/7.5/10%). Additionally, six control samples were prepared—pure milk (M), milk with erythritol (M/E), milk with stevia (M/S), and three samples of 10% aqueous solutions of spice mixtures (10/Cl-Ci, 10/Cl-Tu, and 10/Ci/Tu).

### 4.4. Initial Sample Processing

The beverages and control samples were heated in a water bath (Memmert, type: WNB 7, GmbH, Schwabach, Germany) at 60 °C for 10 min, with constant stirring. The parameters were adjusted to ensure optimal extraction of polyphenols into the milk matrix while protecting them against degradation [87,88,89,90]. Next, the samples were centrifugated at 4 °C at 10,000× *g* for 15 min (MPW-351R, MPW Med, Instruments, Warszawa, Poland), and the supernatants were separated from spice sediments with a nylon sieve. The supernatants were frozen at −20 °C (RB37J5000SA, Samsung Electronics Polska sp. z o.o., Wronki, Poland) until further processing and analyses.

### 4.5. Extraction of Polyphenolic Compounds

Polyphenolic compounds were extracted from the supernatant (1 g) with 0.5 mL of 80% methanol HPLC acidified with 1% HCl (*v/v*). The prepared samples were sonicated twice at 300 W and 40 kHz for 15 min (Sonic 6D, Polsonic, Warsaw, Poland) with a 24 h break in between at 4 °C. The samples were then centrifuged at 14,000× *g* for 6 min, and the supernatants were vacuum-filtered (Samplicity Vacuum Filtration System, Merck, Darmstadt, Germany) through 0.20 µm PTFE hydrophilic membranes (Millex Samplicity Filters, Merck, Darmstadt, Germany). A modified extraction procedure described previously by Oszmiański et al. (2017) [91] was used. The extracts were used for analysis of the antioxidant capacity and total polyphenol content.

### 4.6. Antioxidant Capacity

The antioxidant capacity of the tested beverages and control samples was determined using three methods—FRAP, ABTS, and DPPH. The absorbance of four replicates was measured on Synergy H1 (BioTek, Winooski, VT, USA) against a blank sample of distilled water. The results are expressed as mmoles of Trolox equivalents per 100 g of sample.

#### 4.6.1. FRAP Assay

The FRAP spectrophotometric method measures the ability of an antioxidant to reduce Fe(III) ions to Fe(II) in TPTZ. The colorless reagent produces an intensely blue Fe(II) compound. For the FRAP assay, the method described by Benzie and Strain (1996) [92] with modifications was applied. Briefly, 10 μL of methanolic extract of the sample and 200 μL of FRAP solution (TPTZ) were added. After mixing, the solution was left to stand for 10 min. The absorbance was then measured at a wavelength of λ = 593 nm.

#### 4.6.2. ABTS Assay

The ABTS spectrophotometric method measures the ability of an antioxidant to reduce the blue cation radical ABTS^•+^, manifested by a decrease in the color intensity (absorbance) of the solution. For the ABTS assay, the method described by Re et al. (1999) [93] with some modifications was used. To determine ABTS, 10 μL of methanolic extract of the sample and 200 μL of ABTS solution were added. After mixing, the solution was left to stand for 10 min. The absorbance was then measured at a wavelength of λ = 734 nm.

#### 4.6.3. DPPH Assay

The DPPH spectrophotometric method measures the ability of an antioxidant to reduce the purple radical DPPH^•^, which becomes yellow in its reduced form. DPPH of the samples was determined using the method described by Yen et al. (1995) [94] with some modifications. Briefly, 10 μL of methanolic extract of the sample and 200 μL of DPPH solution were added. After mixing, the solution was left to stand for 10 min. The absorbance was then measured at a wavelength of λ = 517 nm.

### 4.7. Total Polyphenol Content

For the total phenolic content (TPC), the Folin–Ciocalteu method described by Gao et al. (2000) [95] with modifications was applied. This spectrophotometric method is based on the color reaction between the Folin–Ciocalteu reagent and polyphenols in an alkaline environment. By oxidizing phenolic compounds, the yellow Folin–Ciocalteu reagent is reduced and becomes blue. Briefly, 5 μL of methanolic extract of the sample was mixed with 50 μL of 10% sodium carbonate, 100 μL of H_2_O, and 10 μL of Folin–Ciocalteu reagent. After mixing, the solution was left to stand for 1 h. The absorbance of four replicates was then measured at a wavelength of λ = 765 nm on Synergy H1 (BioTek, Winooski, VT, USA). The results are expressed as mg of gallic acid equivalents per 100 g (mg GAE/100 g) of sample.

### 4.8. Wet Mineralization

Wet mineralization was conducted using a modification of the method described previously by Huang et al. (2004) [96]. Briefly, supernatants of beverages (0.6 g ± 0.01 g) were predigested with 7 mL of 65% nitric acid and left standing open for 1 h at room temperature under a fume hood. Next, samples were wet-mineralized using a microwave digestion system MARS 6 (CEM, Warszawa, Poland) at 210 °C and 1800 W for 15 min (program “Food”). After cooling, the mineralizates were quantitatively transferred with redistilled water to 10 mL measuring vessels. Mineralized samples were used for the analysis of mineral element content (Ca, Mg, Fe, Zn, and Cu).

### 4.9. Mineral Element Content

The content of mineral elements—magnesium (Mg), iron (Fe), zinc (Zn), and copper (Cu)—was determined by atomic absorption (emission for calcium (Ca)) spectrometry in an acetylene–oxygen flame (FAAS/FAES) using a spectrometer SpectraAA240 FS (Varian, Agilent Technologies, Palo Alto, Santa Clara, CA, USA). The conditions for measuring mineral elements by FAAS/FAES are presented in Table 9. Measurements of Ca were conducted according to the research procedure PB-06/AAS [97]; Mg according to the standard PN-EN 15505:2009 [98]; and Fe, Zn, and Cu according to the standard 14084:2004 [99].

Mineral element content was calculated using the following formula:

$$mineral\;element\;content\;\left[\frac{mg}{l}\right]=\frac{raw\;spectrometer\;result\;\left[\frac{mg}{l}\right]\;\times \;\;dilution}{\frac{sample\;weight\;[g]}{analyte\;volume\;[ml]}\;\;}$$

The content of Ca and Mg was expressed in mg/100 g of beverage, whereas the content of Fe, Zn, and Cu in µg/100 g.

### 4.10. Statistical Analyses

For statistical analyses, Statistica 13 PL (StatSoft Polska, Kraków, Poland) was employed. The results are expressed as mean ± standard deviation (SD). Three-way ANOVA was carried out to demonstrate the main effects of the type of spice mixture, its share [%], and the type of sweetener, as well as their interactions, on the analyzed features of the tested beverages—antioxidant capacity, total polyphenol content, and mineral element content. Tukey’s HSD test was employed as a post hoc test. Pearson’s correlation was used to verify relationships between the parameters of antioxidant activity, TPC, and mineral element content. Statistical differences between two subgroups (synergistic, antagonistic, and additive effect) were determined using Student’s *t*-test. Differences were considered statistically significant at *p*-value < 0.05 [100].

## 5. Conclusions

The findings of this study clearly demonstrate that the antioxidant potential of functional beverages is significantly influenced by both the type and proportion of spices used, as well as the presence of sweeteners and milk. Beverages with the addition of clove-based mixtures (Cl-Ci and Cl-Tu) exhibited the highest antioxidant activity and total polyphenol content (TPC), confirming the extremely high antioxidant potential of clove and pointing to the value of its use in functional foods counteracting oxidative stress. The increase in antioxidant activity and TPC with increasing spice content observed in this study confirms the contribution of polyphenol-rich spices to the antioxidant potential of functional foods. Erythritol, compared to stevia, was shown to increase both the antioxidant activity and TPC of beverages, most likely due to its higher content in the product weight.

The synergistic effect of spices, milk, and sweeteners seems to be a promising way to increase the antioxidant activity of beverages, which was particularly noticeable in those with added cloves and turmeric (Cl-Tu). However, some combinations of compounds may produce antagonistic effects, as observed in the case of beverages with the addition of cinnamon and turmeric (Ci-Tu). The type of interaction may depend on many physicochemical factors and is difficult to predict theoretically.

The strong positive correlation observed in this study between the share of spice mixtures in tested beverages; the content of iron, copper, and magnesium; and antioxidant activity underscores the functional role of spices as a source of mineral elements.

The high correlation between various antioxidant measurements (FRAP, ABTS, and DPPH) and TPC supports the reliability of the results and their consistency with the existing literature.

Due to the multitude of factors determining the antioxidant activity of a food product (discussed in detail in the Discussion Section), including the combination, concentration, and proportion between the components; complex interactions between them; physicochemical factors; technological processing; and therefore, the difficulty of theoretical prediction—confirmed by the discrepancies in research results—further research is necessary to experimentally develop the composition and technological production process of functional beverages in order to achieve the greatest health-promoting effect. This study highlights the importance of carefully selecting and combining ingredients to maximize the antioxidant properties of functional beverages. Further research is needed to test the properties of beverages produced using combinations of other matrices, spices, and sweeteners, as well as sensory testing with consumers prior to the potential introduction of such functional beverages to the market.

## Figures and Tables

**Table 1:** Three-way ANOVA demonstrating the effect of the type of spice mixture, its share [%], and the type of sweetener and their interactions for the antioxidant capacity (ABTS, FRAP, and DPPH) of tested beverages.

	SS			df	MS			F			*p*		
	ABTS	FRAP	DPPH		ABTS	FRAP	DPPH	ABTS	FRAP	DPPH	ABTS	FRAP	DPPH
Spice mixture	925,913.45	1,098,966.53	296,869.05	2	462,956.72	549,483.26	148,434.52	6978.81	8002.66	1630.08	<0.000	<0.000	<0.000
Share of spice mixture [%]	1,453,763.15	553,273.34	155,128.85	3	484,587.71	184,424.44	51,709.61	7304.88	2685.95	567.86	<0.000	<0.000	<0.000
Sweetener	2014.93	2413.71	3899.39	1	2014.93	2413.71	3899.39	30.37	35.15	42.82	<0.000	<0.000	<0.000
Spice mixture * share of spice mixture [%]	213,038.15	166,834.52	31,892.37	6	35,506.35	27,805.75	5315.39	535.23	404.96	58.37	<0.000	<0.000	<0.000
Spice mixture * sweetener	186.96	2058.26	1485.58	2	93.48	1029.13	742.79	1.40	14.98	8.15	0.251	<0.000	<0.001
Share of spice mixture [%] * sweetener	182.49	1529.49	813.76	3	60.83	509.83	271.25	0.916	7.42	2.97	0.437	<0.001	0.037
Spice mixture * share of spice mixture [%] * sweetener	1851.95	1803.66	1344.61	6	308.65	300.61	224.10	4.65	4.37	2.46	<0.001	<0.001	0.032

SS—type III sum of square, df—degree of freedom, MS—mean square; F—F value, *p*–value of statistical significance. Spice mixture—clove–cinnamon (Cl-Ci)/clove–turmeric (Cl-Tu)/cinnamon–turmeric (Ci-Tu); share of spice mixture—2.5/5/7.5/10 [%]; sweetener—erythritol (E)/ stevia (S); * indicates interaction between factors; statistical significance *p* < 0.05.

**Table 2:** Tukey’s post hoc HSD test for the main effects of the three-way ANOVA demonstrating the effect of the type of spice mixture, its share [%], and the type of sweetener on the antioxidant capacity (ABTS, FRAP, and DPPH) of the tested beverages.

	Mean ± SD			*p*
	ABTS [µM Tx/100 g]	FRAP [µM Tx/100 g]	DPPH [µM Tx/100 g]	
Spice mixture				
Cl-Ci	301.11 ± 159.05 a	261.15 ± 109.90 a	137.72 ± 52.96 a	a,b <0.000
Cl-Tu	305.35 ± 159.13 b	258.00 ± 106.01 b	141.32 ± 57.64 b	
Ci-Tu	94.93 ± 58.63 ab	32.63 ± 18.06 ab	21.59 ± 18.98 ab	
Share of spice mixture [%]				
2.5	111.16 ± 50.17 abc	79.87 ± 49.96 abc	47.26 ± 30.68 abc	a–f <0.000
5.0	176.70 ± 85.44 ade	153.00 ± 95.37 ade	83.31 ± 52.01 ade	
7.5	209.38 ± 89.93 bdf	218.56 ± 128.72 bdf	113.51 ± 69.29 bdf	
10.0	437.94 ± 178.91 cef	284.27 ± 165.61 cef	156.76 ± 80.37 cef	
Sweetener				
E	238.37 ± 164.85	188.94 ± 144.20	106.58 ± 76.98	<0.000
S	229.21 ± 167.75	178.91 ± 134.64	93.83 ± 67.72	

SD—standard deviation, *p*–value of statistical significance; spice mixture—clove–cinnamon (Cl-Ci)/clove–turmeric (Cl-Tu)/cinnamon–turmeric (Ci-Tu); share of spice mixture—2.5/5/7.5/10 [%]; sweetener—erythritol (E)/ stevia (S); a–f—statistically significant differences *p* < 0.05.

**Table 3:** Three-way ANOVA demonstrating the effect of the type of spice mixture, its share [%], and the type of sweetener and their interactions for the total polyphenol content in the tested beverages.

	SS	df	MS	F	*p*
Spice mixture	12,823.25	2	6411.62	573.76	<0.000
Share of spice mixture [%]	10,784.40	3	3594.80	321.69	<0.000
Sweetener	2312.73	1	2312.73	206.96	<0.000
Spice mixture * share of spice mixture [%]	3305.92	6	550.98	49.30	<0.000
Spice mixture * sweetener	691.53	2	345.76	30.94	<0.000
Share of spice mixture [%] * sweetener	426.79	3	142.26	12.73	<0.000
Spice mixture*share of spice mixture [%] * sweetener *	144.08	6	24.01	2.14	0.058

SS—type III sum of square, df—degree of freedom, MS—mean square; F—F value, *p*–value of statistical significance; spice mixture—clove–cinnamon (Cl-Ci)/clove–turmeric (Cl-Tu)/cinnamon–turmeric (Ci-Tu); share of spice mixture—2.5/5/7.5/10 [%]; sweetener—erythritol (E)/ stevia (S); * indicates interaction between factors; statistical significance *p* < 0.05.

**Table 4:** Tukey’s post hoc HSD test for main effects of the three-way ANOVA demonstrating the effect of the type of spice mixture, its share [%], and the type of sweetener on the total polyphenol content of the tested beverages.

TPC [mg GAE/100 g] Mean ± SD								
Spice Mixture			Share of Spice Mixture [%]				Sweetener	
Cl-Ci	Cl-Tu	Ci-Tu	2.5	5	7.5	10	E	S
27.28 ± 15.75 ab	29.49 ± 18.24 bc	3.94 ± 3.87 ac	5.99 ± 4.88 abc	15.93 ± 11.05 ade	24.22 ± 16.80 bdf	34.80 ± 21.52 cef	25.15 ± 20.49 a	15.32 ± 14.04 a

TPC—total polyphenol content; GAE—gallic acid equivalent; spice mixture—clove–cinnamon (Cl-Ci)/ clove–turmeric (Cl-Tu)/ cinnamon–turmeric (Ci-Tu); share of spice mixture—2.5/5/7.5/10 [%]; sweetener—erythritol (E)/ stevia (S)*;* a–f—statistically significant differences *p* < 0.05.

**Table 5:** Three-way ANOVA demonstrating the effect of the type of spice mixture, its share [%], and the type of sweetener and their interactions for the content of mineral elements (Ca, Mg, Fe, Zn, and Cu) of the tested beverages.

	SS					df	MS					F					*p*
	Ca [mg/100 g]	Mg [mg/100 g]	Fe [µg/100 g]	Zn [µg/100 g]	Cu [µg/100 g]		Ca [mg/100 g]	Mg [mg/100 g]	Fe [µg/100 g]	Zn [µg/100 g]	Cu [µg/100 g]	Ca [mg/100 g]	Mg [mg/100 g]	Fe [µg/100 g]	Zn [µg/100 g]	Cu [µg/100 g]	
Spice mixture	1275.2	239.7	310,681.7	14,426.6	25,654.9	2	637.6	119.8	155,340.8	7213.3	12,827.4	175.1	19,509.7	3326.3	505.0	2627.2	<0.000
Share of spice mixture [%]	272.2	1678.5	154,226.0	23,328.6	11,328.3	3	90.7	559.5	51,408.7	7776.2	3776.1	24.9	91,095.2	1100.8	544.4	773.4	<0.000
Sweetener	1524.9	106.4	9986.9	430.2	38,789.6	1	1524.9	106.4	9986.9	430.2	38,789.6	418.8	17,327.3	213.8	30.1	7944.4	<0.000
Spice mixture * share of spice mixture [%]	180.7	61.4	98,116.4	12,900.9	5127.9	6	30.1	10.2	16,352.7	2150.1	854.7	8.3	1666.1	350.2	150.5	175.0	<0.000
Spice mixture * sweetener	501.6	48.8	141,804.0	19,319.8	2243.6	2	250.8	24.4	70,902.0	9659.9	1121.8	68.9	3968.6	1518.2	676.3	229.8	<0.000
Share of spice mixture [%] * sweetener	243.8	4.6	47,417.7	9332.8	2452.4	3	81.3	1.5	15,805.9	3110.9	817.5	22.3	250.9	338.4	217.8	167.4	<0.000
Spice mixture * share of spice mixture [%] * sweetener	268.8	21.7	86,279.4	18,506.1	6819.7	6	44.8	3.6	14,379.9	3084.4	1136.6	12.3	588.0	307.9	215.9	232.8	<0.000

SS—type III sum of square, df—degree of freedom, MS—mean square; F—F value, *p*–value of statistical significance. Spice mixture—clove–cinnamon (Cl-Ci)/clove–turmeric (Cl-Tu)/cinnamon–turmeric (Ci-Tu); share of spice mixture—2.5/5/7.5/10 [%]; sweetener—erythritol (E)/ stevia (S)*;* * indicates interaction between factors; statistical significance *p* < 0.05.

**Table 6:** Tukey’s post hoc HSD test for main effects of three-way ANOVA demonstrating the effect of the type of spice mixture, its share [%], and the type of sweetener on the content of mineral elements (Ca, Mg, Fe, Zn, and Cu) of the tested beverages.

	Mean ± SD					*p*
	Ca [mg/100 g]	Mg [mg/100 g]	Fe [µg/100 g]	Zn [µg/100 g]	Cu [µg/100 g]	
Spice mixture						
Cl-Ci	102.85 ± 7.53 ab	18.90 ± 3.39 ab	784.63 ± 85.28 ab	257.56 ± 43.01 ab	79.87 ± 30.05 ab	a–c 0.000
Cl-Tu	105.77 ± 4.49 ac	22.73 ± 5.46 ac	743.49 ± 64.89 ac	285.54 ± 15.73 ac	57.08 ± 22.45 ac	
Ci-Tu	111.61 ± 5.30 bc	20.32 ± 4.55 bc	648.76 ± 77.30 bc	280.99 ± 25.30 bc	39.96 ± 27.53 bc	
Share of spice mixture [%]						
2.5	109.37 ± 5.96 abc	14.92 ± 1.34 abc	696.99 ± 63.59 ab	257.08 ± 21.17 abc	48.29 ± 28.57 abc	a–f 0.000 [Ca: d 0.028] [Ca: e 0.004]
5.0	106.27 ± 7.99 ad	19.04 ± 1.98 ade	696.98 ± 86.09 cd	266.02 ± 11.32 ade	52.53 ± 24.61 ade	
7.5	106.63 ± 6.28 be	22.36 ± 2.53 bdf	714.63 ± 86.81 ace	276.86 ± 33.87 bdf	58.24 ± 27.99 bdf	
10.0	104.70 ± 6.80 cde	26.27 ± 2.98 cef	793.92 ± 106.50 bde	298.82 ± 39.72 cef	76.75 ± 36.55 cef	
Sweetener						
E	102.76 ± 6.62 a	19.60 ± 4.33 a	735.83 ± 62.39 a	272.58 ± 31.82 a	79.07 ± 14.66 a	a 0.000
S	110.73 ± 4.48 a	21.70 ± 5.00 a	715.43 ± 118.40 a	276.81 ± 33.03 a	38.87 ± 30.56 a	

SD—standard deviation, *p*–value of statistical significance; spice mixture—clove–cinnamon (Cl-Ci)/clove–turmeric (Cl-Tu)/cinnamon–turmeric (Ci-Tu); share of spice mixture—2.5/5/7.5/10 [%]; sweetener—erythritol (E)/stevia (S); a–f—statistically significant differences *p* < 0.05.

**Table 7:** Correlation between antioxidant activity—ABTS, FRAP, DPPH and mineral elements (Ca, Mg, Fe, Zn, and Cu) in the tested beverages.

	ABTS [µM Tx/100 g]		FRAP [µM Tx/100 g]		DPPH [µM Tx/100 g]	
	r	*p*	r	*p*	r	*p*
Ca [mg/100 g]	−0.471	0.0000	−0.539	0.0000	−0.580	0.0000
Mg [mg/100 g]	0.619	0.0000	0.500	0.0000	0.512	0.0000
Fe [µg/100 g]	0.665	0.0000	0.654	0.0000	0.643	0.0000
Zn [µg/100 g]	0.300	0.003	0.143	0.165	0.117	0.257
Cu [µg/100 g]	0.568	0.0000	0.564	0.0000	0.566	0.0000

Statistical significance *p* < 0.05.

**Table 8:** Characteristics of nutritional values and polyphenol content of raw materials used in the production of the tested beverages per 100 g.

Nutrients	Semi-Skimmed (2% Fat) Cow’s Milk	Ground Clove	Ground Cinnamon	Ground Turmeric	Sweetener, Stevia (Glycosides)	Sweetener, Erythritol	Ref
Energy [kcal]	50	274	247	312	0	0	[83] ^1^ [84] ^2^
Macronutrients							
Protein [g]	3.3	6.0	4.0	9.7	0	0	
Fat [g]	2.0	13.0	1.2	3.3	0	0	
Carbohydrates [g]	4.8	65.5	80.6	67.1	100	0	
Mineral elements							
Calcium [mg]	120	632	1000	168	0	nd	
Magnesium [mg]	11	259	60	208	0	nd	
Iron [µg]	20	11,800	8320	55,000	0	nd	
Zinc [µg]	480	2320	1830	4500	0	nd	
Copper [µg]	6	368	339	1300	0	nd	
Phenolic compounds							[85] [86] ^4^
TPC [mg GAE]	0 ^3^	16,047.50	9700.00	2117.00	0 ^3^	0 ^3^	
Phenolic acids [mg]							
Hydroxybenzoic acids							
Gallic acid		458.19					
2-hydroxybenzoic acid			0.70				
Protocatechuic acid		0.52	1.16				
Syringic acid		0.79	0.78				
Hydroxycinnamic acids							
Caffeic acid			24.20				
p-Coumaric acid		8.49	0.55				
Flavonoids [mg]							
Flavonols							
Kaempferol		23.80					
Quercetin		28.40					
Curcuminoids [mg]							
Bisdemethoxycurcumin				1237.50			
Curcumin				2213.57			
Demethoxycurcumin				1982.50			
Other polyphenols [mg]							
Acetyl eugenol		2075.10					
Eugenol		12,592.93					
Cinnamaldehyde		749 ^4^					

^1^ ID numbers in the Food Data Central—for milk, FDC ID: 172205; for ground clove, FDC ID: 171321; for ground cinnamon, FDC ID: 171320; for ground turmeric, FDC ID: 172231; for stevia, FDC ID: 170679; no matched results for erythritol. ^2^ As no data on nutritional value of erythritol was found on USDA Food Data Central, My Food Data was used; nd—no data. ^3^ No data on milk, erythritol, and stevia on Phenol-Explorer; experimental data from this study is presented: TPC of pure milk sample (M), milk with erythritol (M/E), and milk with stevia (M/S) = 0 mg GAE/100 g. ^4^ The content of cinnamaldehyde in Ceylon cinnamon available on the Polish market; in the paper referenced as [86]—Table 1, sample no C20.

**Table 9:** Conditions for determining mineral elements by the FAAS/FAES method.

Parameters	Ca	Mg	Fe	Zn	Cu
Wavelength [nm]	285.2	422.7	372.0	213.9	327.4
Gap [mm]	0.5	0.5	0.2	1.0	0.2
Burner height [cm]	3.0	3.0	3.0	3.0	3.0
Acetylene/oxygen flows [l/h]	70/60	70/60	70/60	70/60	70/60

## Data Availability

Data are placed in the UPWr Research Data Repository at the link https://bazawiedzy.upwr.edu.pl/info/researchdata/UPWRf3b342fbd91a45598d2078f0d0d06a54/ (accessed on 5 September 2025). However, embargo is imposed until all research results are published. Data are available from the corresponding author on reasonable request.

## Supplementary Materials

The following supporting information can be downloaded at https://www.mdpi.com/article/10.3390/ijms26188813/s1.
