# Supplementary material for: Antioxidant Activity, Total Polyphenol Content, and Mineral Composition of Milk Beverages Fortified with Spice Mixtures (Clove, Cinnamon, and Turmeric) and Natural Sweeteners (Erythritol and Stevia): Evidence of Synergistic or Antagonistic Effects of Compounds
Source: Int J Mol Sci. 2025 Sep 10;26(18):8813. doi: 10.3390/ijms26188813 (PMC12469906; doi:10.3390/ijms26188813)
Supplement: Supplementary file 1 [file ijms-26-08813-s001.zip › ijms-3787225-supplementary.pdf]

Table S1. Significance level (*p*) of differences between pairs of the tested beverages and control samples. The results of post-hoc Tukey's HSD test for ABTS (A,D,G), FRAP (B,E,H), and DPPH (C,F,I).

**A**	HSD Tukey's test for ABTS [uM Tx/100g] MS = 147.46, df = 27.000									
	Tested beverages and control samples	{1} 155.21	{2} 231.27	{3} 277.04	{4} 560.22	{5} 141.13	{6} 218.76	{7} 262.96	{8} 562.26	{9} 467.36
1	M/E/Cl-Ci/2.5		0.000151	0.000151	0.000151	0.775435	0.000152	0.000151	0.000151	0.000151
2	M/E/Cl-Ci/5	0.000151		0.000497	0.000151	0.000151	0.865300	0.023644	0.000151	0.000151
3	M/E/Cl-Ci/7.5	0.000151	0.000497		0.000151	0.000151	0.000156	0.775435	0.000151	0.000151
4	M/E/Cl-Ci/10	0.000151	0.000151	0.000151		0.000151	0.000151	0.000151	1.000000	0.000151
5	M/S/Cl-Ci/2.5	0.775435	0.000151	0.000151	0.000151		0.000151	0.000151	0.000151	0.000151
6	M/S/Cl-Ci/5	0.000152	0.865300	0.000156	0.000151	0.000151		0.000716	0.000151	0.000151
7	M/S/Cl-Ci/7.5	0.000151	0.023644	0.775435	0.000151	0.000151	0.000716		0.000151	0.000151
8	M/S/Cl-Ci/10	0.000151	0.000151	0.000151	1.000000	0.000151	0.000151	0.000151		0.000151
9	aq/Cl-Ci/10	0.000151	0.000151	0.000151	0.000151	0.000151	0.000151	0.000151	0.000151	

**B**	HSD Tukey's test for FRAP [uM Tx/100g] MS = 170.04, df = 27.000									
	Tested beverages and control samples	{1} 115.55	{2} 210.39	{3} 320.57	{4} 415.97	{5} 118.83	{6} 218.65	{7} 291.68	{8} 397.55	{9} 344.25
1	M/E/Cl-Ci/2.5		0.000151	0.000151	0.000151	0.999990	0.000151	0.000151	0.000151	0.000151
2	M/E/Cl-Ci/5	0.000151		0.000151	0.000151	0.000151	0.991377	0.000151	0.000151	0.000151
3	M/E/Cl-Ci/7.5	0.000151	0.000151		0.000151	0.000151	0.000151	0.082738	0.000152	0.245643
4	M/E/Cl-Ci/10	0.000151	0.000151	0.000151		0.000151	0.000151	0.000151	0.557463	0.000152
5	M/S/Cl-Ci/2.5	0.999990	0.000151	0.000151	0.000151		0.000151	0.000151	0.000151	0.000151
6	M/S/Cl-Ci/5	0.000151	0.991377	0.000151	0.000151	0.000151		0.000152	0.000151	0.000151
7	M/S/Cl-Ci/7.5	0.000151	0.000151	0.082738	0.000151	0.000151	0.000152		0.000151	0.000275
8	M/S/Cl-Ci/10	0.000151	0.000151	0.000152	0.557463	0.000151	0.000151	0.000151		0.000247
9	aq/Cl-Ci/10	0.000151	0.000151	0.245643	0.000152	0.000151	0.000151	0.000275	0.000247	

**C**	HSD Tukey's test for DPPH [uM Tx/100g] MS = 118.67, df = 27.000									
	Tested beverages and control samples	{1} 75.181	{2} 119.52	{3} 169.16	{4} 228.44	{5} 69.676	{6} 113.42	{7} 139.87	{8} 186.46	{9} 206.71
1	M/E/Cl-Ci/2.5		0.000254	0.000151	0.000151	0.998160	0.001073	0.000151	0.000151	0.000151
2	M/E/Cl-Ci/5	0.000254		0.000165	0.000151	0.000164	0.996245	0.216165	0.000151	0.000151
3	M/E/Cl-Ci/7.5	0.000151	0.000165		0.000152	0.000151	0.000153	0.018138	0.406642	0.001325
4	M/E/Cl-Ci/10	0.000151	0.000151	0.000152		0.000151	0.000151	0.000151	0.000398	0.155403
5	M/S/Cl-Ci/2.5	0.998160	0.000164	0.000151	0.000151		0.000283	0.000151	0.000151	0.000151
6	M/S/Cl-Ci/5	0.001073	0.996245	0.000153	0.000151	0.000283		0.042964	0.000151	0.000151
7	M/S/Cl-Ci/7.5	0.000151	0.216165	0.018138	0.000151	0.000151	0.042964		0.000195	0.000151
8	M/S/Cl-Ci/10	0.000151	0.000151	0.406642	0.000398	0.000151	0.000151	0.000195		0.221258
9	aq/Cl-Ci/10	0.000151	0.000151	0.001325	0.155403	0.000151	0.000151	0.000151	0.221258	

D	HSD Tukey’s test for ABTS [uM Tx/100g] MS = 136.4, df = 27.000									
	Tested beverages and control samples	{1} 155.21	{2} 248.73	{3} 280.74	{4} 561.32	{5} 128.00	{6} 242.28	{7} 263.44	{8} 563.05	{9} 288.04
1	M/E/Cl-Tu/2.5		0.000151	0.000151	0.000151	0.058449	0.000151	0.000151	0.000151	0.000151
2	M/E/Cl-Tu/5	0.000151		0.015234	0.000151	0.000151	0.996574	0.693510	0.000151	0.001750
3	M/E/Cl-Tu/7.5	0.000151	0.015234		0.000151	0.000151	0.002246	0.496832	0.000151	0.992089
4	M/E/Cl-Tu/10	0.000151	0.000151	0.000151		0.000151	0.000151	0.000151	1.000000	0.000151
5	M/S/Cl-Tu/2.5	0.058449	0.000151	0.000151	0.000151		0.000151	0.000151	0.000151	0.000151
6	M/S/Cl-Tu/5	0.000151	0.996574	0.002246	0.000151	0.000151		0.248489	0.000151	0.000344
7	M/S/Cl-Tu/7.5	0.000151	0.693510	0.496832	0.000151	0.000151	0.248489		0.000151	0.113764
8	M/S/Cl-Tu/10	0.000151	0.000151	0.000151	1.000000	0.000151	0.000151	0.000151		0.000151
9	aq/Cl-Tu/10	0.000151	0.001750	0.992089	0.000151	0.000151	0.000344	0.113764	0.000151	

E	HSD Tukey’s test for FRAP [uM Tx/100g] MS = 79.809, df = 27.000									
	Tested beverages and control samples	{1} 118.63	{2} 229.68	{3} 329.81	{4} 397.65	{5} 103.80	{6} 216.55	{7} 285.36	{8} 382.56	{9} 232.89
1	M/E/Cl-Tu/2.5		0.000151	0.000151	0.000151	0.350632	0.000151	0.000151	0.000151	0.000151
2	M/E/Cl-Tu/5	0.000151		0.000151	0.000151	0.000151	0.506312	0.000151	0.000151	0.999849
3	M/E/Cl-Tu/7.5	0.000151	0.000151		0.000151	0.000151	0.000151	0.000154	0.000152	0.000151
4	M/E/Cl-Tu/10	0.000151	0.000151	0.000151		0.000151	0.000151	0.000151	0.329536	0.000151
5	M/S/Cl-Tu/2.5	0.350632	0.000151	0.000151	0.000151		0.000151	0.000151	0.000151	0.000151
6	M/S/Cl-Tu/5	0.000151	0.506312	0.000151	0.000151	0.000151		0.000151	0.000151	0.237815
7	M/S/Cl-Tu/7.5	0.000151	0.000151	0.000154	0.000151	0.000151	0.000151		0.000151	0.000152
8	M/S/Cl-Tu/10	0.000151	0.000151	0.000152	0.329536	0.000151	0.000151	0.000151		0.000151
9	aq/Cl-Tu/10	0.000151	0.999849	0.000151	0.000151	0.000151	0.237815	0.000152	0.000151	

F	HSD Tukey’s test for DPPH [uM Tx/100g] MS = 47.393, df = 27.000									
	Tested beverages and control samples	{1} 70.266	{2} 127.77	{3} 177.32	{4} 220.87	{5} 56.208	{6} 114.90	{7} 155.01	{8} 208.19	{9} 146.48
1	M/E/Cl-Tu/2.5		0.000151	0.000151	0.000151	0.136522	0.000151	0.000151	0.000151	0.000151
2	M/E/Cl-Tu/5	0.000151		0.000151	0.000151	0.000151	0.214727	0.000318	0.000151	0.016534
3	M/E/Cl-Tu/7.5	0.000151	0.000151		0.000151	0.000151	0.000151	0.002677	0.000170	0.000170
4	M/E/Cl-Tu/10	0.000151	0.000151	0.000151		0.000151	0.000151	0.000151	0.230546	0.000151
5	M/S/Cl-Tu/2.5	0.136522	0.000151	0.000151	0.000151		0.000151	0.000151	0.000151	0.000151
6	M/S/Cl-Tu/5	0.000151	0.214727	0.000151	0.000151	0.000151		0.000152	0.000151	0.000163
7	M/S/Cl-Tu/7.5	0.000151	0.000318	0.002677	0.000151	0.000151	0.000152		0.000151	0.710732
8	M/S/Cl-Tu/10	0.000151	0.000151	0.000170	0.230546	0.000151	0.000151	0.000151		0.000151
9	aq/Cl-Tu/10	0.000151	0.016534	0.000170	0.000151	0.000151	0.000163	0.710732	0.000151	

G	HSD Tukey's test for ABTS [uM Tx/100g] MS = 50.18, df = 27.000									
	Tested beverages and control samples	{1} 41.092	{2} 65.395	{3} 85.057	{4} 199.21	{5} 46.283	{6} 53.754	{7} 87.023	{8} 181.60	{9} 209.39
1	M/E/Ci-Tu/2.5		0.001403	0.000151	0.000151	0.978458	0.263159	0.000151	0.000151	0.000151
2	M/E/Ci-Tu/5	0.001403		0.013515	0.000151	0.017608	0.363433	0.005156	0.000151	0.000151
3	M/E/Ci-Tu/7.5	0.000151	0.013515		0.000151	0.000152	0.000176	0.999978	0.000151	0.000151
4	M/E/Ci-Tu/10	0.000151	0.000151	0.000151		0.000151	0.000151	0.000151	0.035473	0.536309
5	M/S/Ci-Tu/2.5	0.978458	0.017608	0.000152	0.000151		0.849748	0.000152	0.000151	0.000151
6	M/S/Ci-Tu/5	0.263159	0.363433	0.000176	0.000151	0.849748		0.000159	0.000151	0.000151
7	M/S/Ci-Tu/7.5	0.000151	0.005156	0.999978	0.000151	0.000152	0.000159		0.000151	0.000151
8	M/S/Ci-Tu/10	0.000151	0.000151	0.000151	0.035473	0.000151	0.000151	0.000151		0.000340
9	aq/Ci-Tu/10	0.000151	0.000151	0.000151	0.536309	0.000151	0.000151	0.000151	0.000340	

H	HSD Tukey's test for FRAP [uM Tx/100g] MS = 13.668, df = 27.000									
	Tested beverages and control samples	{1} 13.169	{2} 20.867	{3} 38.931	{4} 56.072	{5} 9.2178	{6} 21.893	{7} 45.038	{8} 55.815	{9} 127.69
1	M/E/Ci-Tu/2.5		0.121972	0.000151	0.000151	0.840580	0.053249	0.000151	0.000151	0.000151
2	M/E/Ci-Tu/5	0.121972		0.000155	0.000151	0.003663	0.999978	0.000151	0.000151	0.000151
3	M/E/Ci-Tu/7.5	0.000151	0.000155		0.000161	0.000151	0.000162	0.356871	0.000164	0.000151
4	M/E/Ci-Tu/10	0.000151	0.000151	0.000161		0.000151	0.000151	0.006558	1.000000	0.000151
5	M/S/Ci-Tu/2.5	0.840580	0.003663	0.000151	0.000151		0.001412	0.000151	0.000151	0.000151
6	M/S/Ci-Tu/5	0.053249	0.999978	0.000162	0.000151	0.001412		0.000151	0.000151	0.000151
7	M/S/Ci-Tu/7.5	0.000151	0.000151	0.356871	0.006558	0.000151	0.000151		0.008349	0.000151
8	M/S/Ci-Tu/10	0.000151	0.000151	0.000164	1.000000	0.000151	0.000151	0.008349		0.000151
9	aq/Ci-Tu/10	0.000151	0.000151	0.000151	0.000151	0.000151	0.000151	0.000151	0.000151	

I	HSD Tukey's test for DPPH [uM Tx/100g] MS = 100.29, df = 27.000									
	Tested beverages and control samples	{1} 5.4819	{2} 14.428	{3} 22.882	{4} 47.655	{5} 6.7599	{6} 9.8074	{7} 16.793	{8} 48.933	{9} 89.572
1	M/E/Ci-Tu/2.5		0.933509	0.295594	0.000209	1.000000	0.999408	0.798050	0.000185	0.000151
2	M/E/Ci-Tu/5	0.933509		0.951031	0.002062	0.971995	0.999049	0.999994	0.001331	0.000151
3	M/E/Ci-Tu/7.5	0.295594	0.951031		0.037026	0.389315	0.653110	0.993414	0.024342	0.000151
4	M/E/Ci-Tu/10	0.000209	0.002062	0.037026		0.000248	0.000485	0.004663	1.000000	0.000215
5	M/S/Ci-Tu/2.5	1.000000	0.971995	0.389315	0.000248		0.999956	0.881598	0.000209	0.000151
6	M/S/Ci-Tu/5	0.999408	0.999049	0.653110	0.000485	0.999956		0.984077	0.000352	0.000151
7	M/S/Ci-Tu/7.5	0.798050	0.999994	0.993414	0.004663	0.881598	0.984077		0.002991	0.000151
8	M/S/Ci-Tu/10	0.000185	0.001331	0.024342	1.000000	0.000209	0.000352	0.002991		0.000259
9	aq/Ci-Tu/10	0.000151	0.000151	0.000151	0.000215	0.000151	0.000151	0.000151	0.000259	

Table S2. Significance level (p) of differences between pairs of the tested beverages and control samples. The results of post-hoc Tukey's HSD test for total polyphenol content (TPC)

**A**	HSD Tukey's test for TPC [mg GAE/100g] MS = 11.993, df = 27.000									
	Tested beverages and control samples	{1} 11.045	{2} 24.969	{3} 40.179	{4} 52.994	{5} 5.5023	{6} 15.480	{7} 27.497	{8} 40.534	{9} 266.97
1	M/E/Cl-Ci/2.5		0.000281	0.000151	0.000151	0.396724	0.675125	0.000157	0.000151	0.000151
2	M/E/Cl-Ci/5	0.000281		0.000178	0.000151	0.000152	0.015255	0.978971	0.000169	0.000151
3	M/E/Cl-Ci/7.5	0.000151	0.000178		0.000601	0.000151	0.000151	0.000671	1.000000	0.000151
4	M/E/Cl-Ci/10	0.000151	0.000151	0.000601		0.000151	0.000151	0.000151	0.000813	0.000151
5	M/S/Cl-Ci/2.5	0.396724	0.000152	0.000151	0.000151		0.009393	0.000151	0.000151	0.000151
6	M/S/Cl-Ci/5	0.675125	0.015255	0.000151	0.000151	0.009393		0.001224	0.000151	0.000151
7	M/S/Cl-Ci/7.5	0.000157	0.978971	0.000671	0.000151	0.000151	0.001224		0.000504	0.000151
8	M/S/Cl-Ci/10	0.000151	0.000169	1.000000	0.000813	0.000151	0.000151	0.000504		0.000151
9	aq/Cl-Ci/10	0.000151	0.000151	0.000151	0.000151	0.000151	0.000151	0.000151	0.000151	

**B**	HSD Tukey's test for TPC [mg GAE/100g] MS = 23.160, df = 27.000									
	Tested beverages and control samples	{1} 12.420	{2} 30.202	{3} 45.589	{4} 62.307	{5} 3.8173	{6} 20.047	{7} 25.634	{8} 35.922	{9} 157.88
1	M/E/Cl-Tu/2.5		0.000610	0.000151	0.000151	0.263142	0.409390	0.014965	0.000155	0.000151
2	M/E/Cl-Tu/5	0.000610		0.003119	0.000151	0.000152	0.112670	0.909136	0.752307	0.000151
3	M/E/Cl-Tu/7.5	0.000151	0.003119		0.001209	0.000151	0.000152	0.000226	0.149580	0.000151
4	M/E/Cl-Tu/10	0.000151	0.000151	0.001209		0.000151	0.000151	0.000151	0.000152	0.000151
5	M/S/Cl-Tu/2.5	0.263142	0.000152	0.000151	0.000151		0.001712	0.000166	0.000151	0.000151
6	M/S/Cl-Tu/5	0.409390	0.112670	0.000152	0.000151	0.001712		0.774144	0.002201	0.000151
7	M/S/Cl-Tu/7.5	0.014965	0.909136	0.000226	0.000151	0.000166	0.774144		0.104055	0.000151
8	M/S/Cl-Tu/10	0.000155	0.752307	0.149580	0.000152	0.000151	0.002201	0.104055		0.000151
9	aq/Cl-Tu/10	0.000151	0.000151	0.000151	0.000151	0.000151	0.000151	0.000151	0.000151	

**C**	HSD Tukey's test for TPC [mg GAE/100g] MS = 9.1826, df = 27.000									
	Tested beverages and control samples	{1} 1.8430	{2} 3.1078	{3} 4.3050	{4} 12.775	{5} 1.3340	{6} 1.7774	{7} 2.1322	{8} 4.2607	{9} 102.01
1	M/E/Ci-Tu/2.5		0.999539	0.960514	0.000790	1.000000	1.000000	1.000000	0.964412	0.000151
2	M/E/Ci-Tu/5	0.999539		0.999691	0.003198	0.994901	0.999334	0.999933	0.999766	0.000151
3	M/E/Ci-Tu/7.5	0.960514	0.999691		0.012648	0.893300	0.954199	0.981124	1.000000	0.000151
4	M/E/Ci-Tu/10	0.000790	0.003198	0.012648		0.000490	0.000740	0.001067	0.012029	0.000151
5	M/S/Ci-Tu/2.5	1.000000	0.994901	0.893300	0.000490		1.000000	0.999986	0.900878	0.000151
6	M/S/Ci-Tu/5	1.000000	0.999334	0.954199	0.000740	1.000000		1.000000	0.958546	0.000151
7	M/S/Ci-Tu/7.5	1.000000	0.999933	0.981124	0.001067	0.999986	1.000000		0.983378	0.000151
8	M/S/Ci-Tu/10	0.964412	0.999766	1.000000	0.012029	0.900878	0.958546	0.983378		0.000151
9	aq/Ci-Tu/10	0.000151	0.000151	0.000151	0.000151	0.000151	0.000151	0.000151	0.000151	
